# The Use of Kernel Density Estimation With a Bio-Physical Model Provides a Method to Quantify Connectivity Among Salmon Farms: Spatial Planning and Management With Epidemiological Relevance

**DOI:** 10.3389/fvets.2018.00269

**Published:** 2018-10-30

**Authors:** Danielle L. Cantrell, Erin E. Rees, Raphael Vanderstichel, Jon Grant, Ramón Filgueira, Crawford W. Revie

**Affiliations:** ^1^Department of Health Management, Atlantic Veterinary College, University of Prince Edward Island, Charlottetown, PE, Canada; ^2^Land and Sea Systems Analysis, Granby, QC, Canada; ^3^Department of Oceanography, Dalhousie University, Halifax, NS, Canada; ^4^Marine Affairs Program, Dalhousie University, Halifax, NS, Canada

**Keywords:** aquatic epidemiology, sea lice, salmon lice, kernel density, infectious pressure, disease networks, marine spatial planning, connectivity

## Abstract

Connectivity in an aquatic setting is determined by a combination of hydrodynamic circulation and the biology of the organisms driving linkages. These complex processes can be simulated in coupled biological-physical models. The physical model refers to an underlying circulation model defined by spatially-explicit nodes, often incorporating a particle-tracking model. The particles can then be given biological parameters or behaviors (such as maturity and/or survivability rates, diel vertical migrations, avoidance, or seeking behaviors). The output of the bio-physical models can then be used to quantify connectivity among the nodes emitting and/or receiving the particles. Here we propose a method that makes use of kernel density estimation (KDE) on the output of a particle-tracking model, to quantify the infection or infestation pressure (IP) that each node causes on the surrounding area. Because IP is the product of both exposure time and the concentration of infectious agent particles, using KDE (which also combine elements of time and space), more accurately captures IP. This method is especially useful for those interested in infectious agent networks, a situation where IP is a superior measure of connectivity than the probability of particles from each node reaching other nodes. Here we illustrate the method by modeling the connectivity of salmon farms via sea lice larvae in the Broughton Archipelago, British Columbia, Canada. Analysis revealed evidence of two sub-networks of farms connected via a single farm, and evidence that the highest IP from a given emitting farm was often tens of kilometers or more away from that farm. We also classified farms as net emitters, receivers, or balanced, based on their structural role within the network. By better understanding how these salmon farms are connected to each other via their sea lice larvae, we can effectively focus management efforts to minimize the spread of sea lice between farms, advise on future site locations and coordinated treatment efforts, and minimize any impact of farms on juvenile wild salmon. The method has wide applicability for any system where capturing infectious agent networks can provide useful guidance for management or preventative planning decisions.

## Introduction

Marine ecosystems are connected by hydrodynamic exchange through linked parcels of water. Understanding the various and complex ways in which these connections happen over space and time is the domain of connectivity modeling. The scale of the question can alter the definition of connectivity, but in its broadest sense, connectivity is the exchange of individuals among geographically separated subpopulations of a meta-population (1). In contrast to the terrestrial case, marine connectivity has the additional complication of operating on larger spatial scales, due to fewer dispersal barriers and an ideal medium for transporting larvae, viruses, or other agents over large distances (2). Marine connectivity studies have been used to investigate questions across multiple disciplines. For example, for planning of marine protected areas based on planktonic connectivity (3, 4), to understand how coral reefs are connected via gametes (5), to determine best release locations for sea turtle hatchlings (6), for spatial planning of salmon farm locations (7), and to determine the ideal groups of farms for coordinated treatments to prevent disease spread among salmon farms (8, 9). Connectivity in marine systems is driven in large part by the underlying circulation of the system of interest; thus abiotic factors such as wind or storm events, river discharge, tidal cycles, and heat fluxes can impact connectivity (10–12). Likewise, the behavior and/or life history of the organism of interest driving the connectivity is important. For example, passive viral particles will be almost exclusively driven by circulation (11). This is in contrast to sea turtle hatchlings or planktonic larvae, which may swim to avoid or seek certain conditions, have species-specific energy reserves, and maturation rates which can be impacted by the abiotic conditions (6).

To account for the complexity of the many drivers of connectivity in aquatic systems, coupled models which link biology and physical (bio-physical) modeling are increasingly being used (3–6, 11, 13–15). In this approach, an underlying circulation model using a framework such as the Finite-Volume Community Ocean Model (FVCOM) or Regional Ocean Modeling System (ROMS) is first developed. Then, the output from the circulation model is used to inform a computerized particle-tracking model. The particles, in turn, have a biological model associated with them. Processes such as mortality and maturity, swimming avoidance behaviors, or diel vertical migration can be determined for each particle by exposure to certain abiotic criteria such as temperature and salinity. Output from bio-physical models can then be used to quantify spatiotemporal patterns in connectivity of the aquatic ecosystem.

In the case of infectious agent connectivity, there is an additional challenge related to the uncertainty on how to probabilistically link the physical presence of the agent to the biological implications of its presence, that is, effective infection or infestation pressure (IP). IP has been used in deterministic models to estimate the influence each source of infectious agents exerts on the surrounding area (16–18). IP is a measure of a combination of the amount of infectious agents present and the exposure time near a susceptible host (17). In the context of infectious diseases, where the outcome of interest is illness or infestation, IP is an ideal measure to quantify the impact of point sources of infectious agents. While IP has been used in deterministic modeling (17, 19), it has not, to our knowledge, been used in the context of network or connectivity models that are based on bio-physical simulation output. Rather, recent biophysical modeling of sea lice dispersal in such areas as Norway, Scotland, and the Faroe islands have used a probabilistic approach to estimate connectivity between farms (7, 15, 20, 21).

Here we illustrate the use of kernel density estimation (KDE) in quantifying connectivity from bio-physical modeling outputs via IP estimates. KDE is a technique with a full and robust literature, and has been used widely in ecology, epidemiology, sociology, etc. since the 1950s (22). Briefly, it is a non-parametric technique for estimating the underlying distribution, or probability density function (PDF) of a continuous, non-random variable. It does this by making a smoothed “kernel” centered around each datum, then adds them to estimate the PDF of the data as a whole (23). Our method uses KDE performed on snapshots (i.e., cross-sections of time) of particle locations to determine the IP each node (i.e., the spatially explicit source for each cohort of particles) exerts on all the other nodes. This approach aims to estimate connectivity as a factor of both time and infectious dose. Taking into account the amount of time a particle spends in a given location is important in infectious agent connectivity studies, as the longer the potential hosts are exposed to the infectious particles (exposure time), the more likely it is that this will result in an infection/disease or infestation (24, 25). Thus, our approach is particularly suitable to measure connectivity of infectious agent networks.

We have chosen a salmon farming area as our “test” system to apply this KDE method to estimate IP approach: the Broughton Archipelago (BA) in British Columbia (BC), Canada. The BA was chosen because in addition to having active salmon farming in the region, it is also an area where many major rivers from BC meet the ocean, and thus is home to some large and ecologically important juvenile wild salmon out-migrations to the sea (12). Thus, it is important economically as well as from a conservation perspective. We have elected to model sea lice dispersion because sea lice are one of the most costly and persistent problems facing salmon farmers as well as one of the most concerning threats to wild salmonids from farmed salmon (26–29). Additionally, sea lice have a planktonic stage before they become infectious, and can be carried tens of km away from where they are released before they attach to a host (24, 30, 31). Thus, sea lice larvae IP estimates from our approach are likely to yield results that are not intuitive based simply on sea-way distance measures.

*Lepeoptheirus salmonis* is the species of most concern to salmon farming operations in the northern hemisphere. The *L. salmonis* louse has two planktonic nauplii stages before molting to an infective copepod stage, at which point it can attach to a host (24, 31), and consume their skin and mucus(32, 33). After attachment, there are two chalimus stages, two pre-adult stages, and finally an adult stage. The development times at nearly every stage are temperature dependent. Females can produce egg strings of upwards of 300 eggs each after mating, depending on what temperature the females are exposed to during egg production (34, 35). These eggs require between 17.5 days at 5°C and 5.5 days at 15°C to hatch (36). Development time to the infectious copepod stage ranges between 10 days at 5°C to about 2 days at 15°C (37–40). Additionally, while there is disagreement in the literature over the exact level at which low salinity begins to impact sea lice larvae survival, (37, 38, 40, 41), there is agreement that salinity has an important impact. Thus, the complex and high plasticity life cycle of sea lice demands a biological model.

This sea lice/salmon farm biological-system is important because the potential for sea lice to spill over into wild salmon populations is a major conservation issue worldwide (42–48). Sea lice attachment to adult fish can cause lesions, making them susceptible to secondary infections, and also results in reduced feeding. The consequences for juvenile salmon can include death due to osmoregulatory failure (32, 33, 41, 49). Additionally, the complexity of management issues for controlling sea lice infestation make understanding disease dynamics and farm-to-farm connectivity via infective sea lice larvae crucial in terms of: sustainability of the industry, reduction in the number of sea lice treatments, acceptance by the public, and minimizing impact on wild salmonids. Furthermore, increased welfare/decreased stress of farmed fish could lead to reduced outbreaks of other diseases at farms (39, 50–55).

Our goal is to establish a new methodology for using bio-physical modeling outputs. Because the results of this bio-physical output remain unvalidated [though the physical model is validated in Foreman et al. (10)], any management suggestions/implications should be used more for hypothesis generation than immediate implementation into management. While not shown in this paper, validation using real sea lice counts and sensitivity analysis around some of the simulation parameters is the focus of a forthcoming paper. Here we assess data from a bio-physical particle tracking circulation model for the BA region run over a 5 month window (March 3rd–July 30th, 2009) chosen to coincide with the juvenile wild salmon outmigration period (56). The biological model has equations governing the maturation of individual particles based on the temperatures the particles are exposed to, and survivability based on exposure to varying salinity. The physical model refers to the underlying FVCOM circulation model, which includes the river discharge to capture the important spring freshet event. We apply a KDE method to estimate the infestation pressure each farm has on its neighbors and characterize the network of farms during the simulation period. We make recommendations for coordinated treatment based on the network results of the “baseline” connectivity (i.e. the connectivity outside of stochastic events that lead to temporary spikes in overall connectivity of the network), and then discuss the potential applications of using our KDE approach in the study of aquatic epidemiology.

## Methods

### Study area

**Figure 1:**
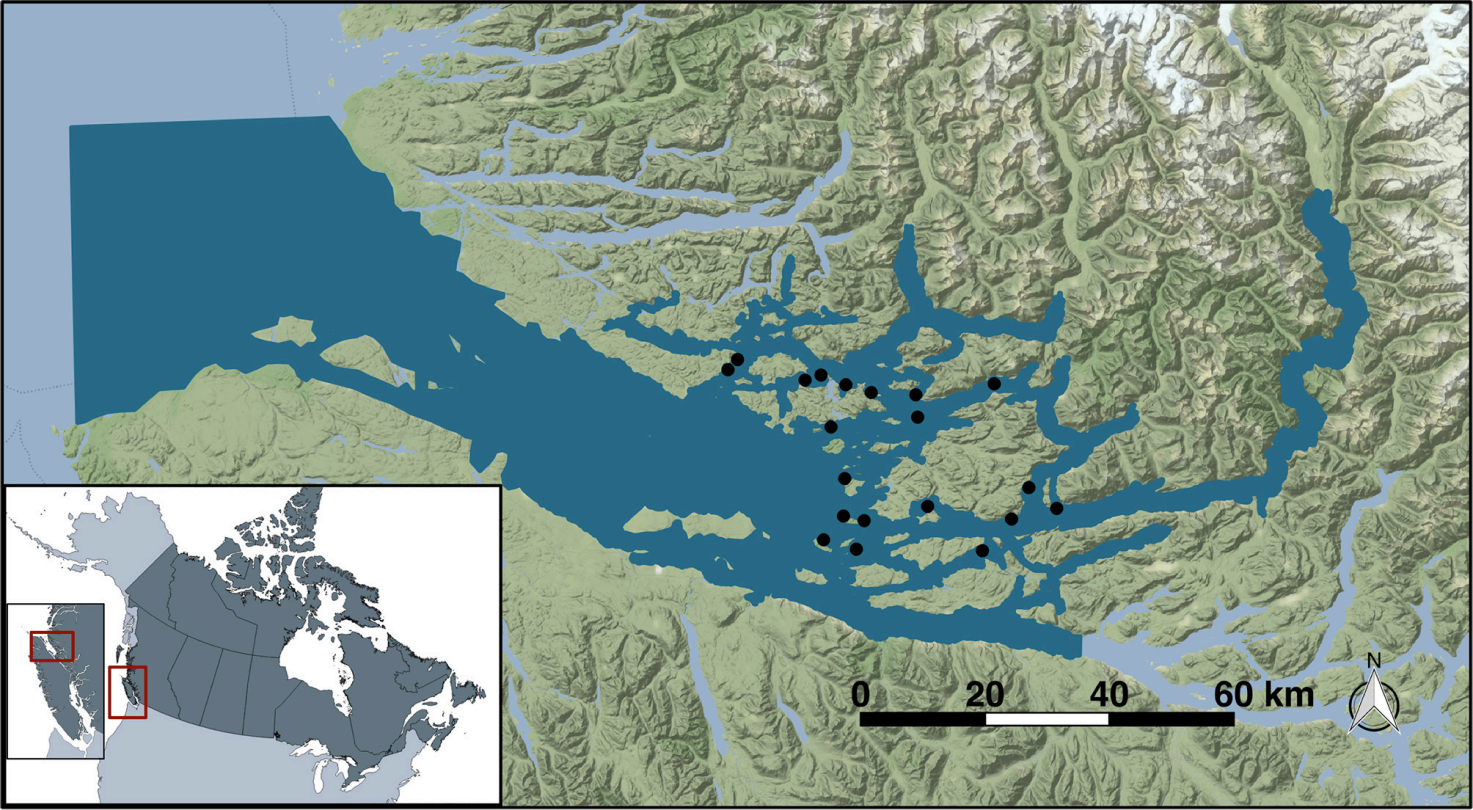
Study location of Broughton Archipelago (red inset) in British Columbia. Dark blue illustrates the extent of the model domain while the study farm sites are indicated as black dots.

The Broughton Archipelago is a group of islands off the northeastern tip of Vancouver Island, in the northeastern flank of the Queen Charlotte Strait on the coast of British Columbia, Canada (Figure 1). This area has around 20 active salmon farms and is also home to important juvenile wild salmon populations. As such, it is both important commercially and as an area with ongoing conservation efforts. Salmon farms are managed so as to minimize the likelihood of juvenile salmon encountering sea lice larvae during their outmigration (13).

### Simulation period

The simulation used in this study ran from March 3rd to July 31st (57). The circulation model mimics conditions from 2009. The FVCOM model began the simulation on March 1st, 2009. FVCOM takes around 8 days to ramp up (Mike Foreman, personal communication), so it was allowed 10 days to initialize as a precautionary approach. Thus the first day of the particle-tracking model was March 11th, 2009. The particles were all tracked for 11 days following their release. Thus, the last particle releases occurred on July 20th, but the simulation ran until July 31st.

### FVCOM model

For the underlying circulation model, the Finite Volume Coastal Oceanographic Model (FVCOM) was used. More details and validation of this model can be found in Foreman et al. (10). Briefly, the FVCOM model uses an unstructured, triangular grid to organize simulated processes (13). This allows higher resolution in complex topography and bathymetry, and less resolution in wide channels. The grid for our model domain had 42,682 junctions and 74,774 triangles. Triangles' sides varied in length from ~2.3 km in wide channels to <50 m in narrow passages. There were 21 vertical gridded layers with variable spacing from sea bottom to surface, with the highest resolution at the surface and bottom layers, and maximum inter layer spacing of 9% of the depth at mid column. Depth ranged between 3 and 520 m, resulting in a possible maximum vertical grid layer size ranging between 27 cm and 46.8 m at mid-column, depending on the depth of the grid. Freshwater discharge data from the six major rivers in the BA were used for this forcing (10).

### Particle tracking model

The particle tracking model simulated the release of 50 particles from each farm every hour for March 11th–July 20th, 2009; a total of 130 days. One single release of particles across all 20 farms was termed a “pulse.” The location of every particle once released was recorded every 20 min, and every pulse was followed for 11 days post release. The 11 days was a reasonable assumption based on real mean sea surface temperatures for the BA during the simulation period, which ranges between 7.4 and 10.1 C, which corresponds to 6.1 and 3.6 days, respectively, until maturation (based on the maturation formula used from (36), and explained below) (12, 54). The max constraint of 11 days was chosen as a sea louse is not likely to live past this time frame if they are not by then attached to a host, due to limited energy reserves (58). However, this could underestimate long distance dispersal pathways for particles that are exposed to cold water temperatures (i.e. winter conditions in the BA, or particles that may encounter deeper, colder water).Thus, there is potential for some particles to be infective passed the 11 day window we chose (35). In particular, future studies looking at model domains larger than our current model domain (roughly 85 km from north to south), such as modeling the entire coast of British Columbia, should consider increasing the tracking time of the particles.

Particle movements were dictated by a combination of the underlying circulation model and a random walk component, computed by the following equation:

(1) $$\begin{array}{r} {\text{L}}^{\text{t}}\left(\text{x},\text{y},\text{z}\right)={\text{L}}^{\text{t}-\text{Δt}}\left(\text{x},\text{y},\text{z}\right)+\text{Δ}\text{t}\left[\text{U}\left(\text{x},\text{y},\text{z}\right)\right]+\delta \text{H}\left(\text{x},\text{y}\right)+\delta \text{z}\left(\text{z}\right) \end{array}$$

Where, L^t^(x, y, z) is the location of a particle at time t, Δt is the time step of particle tracking algorithm (60 s for this simulation), U(x, y, z) is the velocity from circulation model, and δH and δz represent the horizontal and vertical random walk adjustments to the particle position, respectively. Model velocities were linearly interpolated in space and time and the particle's advection was calculated using a 4th order Runge–Kutta algorithm. As sea lice larvae use their limited swimming abilities to remain in the upper few meters of the water column (59, 60), particles were constrained to remain in the top 5 m (61). Particles were prevented from becoming grounded on the shoreline by holding their positions when a trajectory predicted stranding. The positions of the particles were held until the velocity field of a new time step in the model carried them away from land (12).

### Biological model

The particles also had a biological model associated with them which calculated maturity and survivability based on temperature and salinity of the water the particles were exposed to and the age of the particle. Particles were released as “pre-infectious” nauplii (I or II), and molted into infectious copepods. Pre-infectious particles have a maturity value < 1, and infectious particles have a maturity value ≥ 1. The development time (time until the particle molts to a copepod, i.e., when maturity = 1) was calculated based on temperature, and was modeled using a simplified Bělehrádek function (36).

(2) $$\begin{array}{r} \tau \left(T\right)={\left[\frac{\beta_{1}}{T-10+\beta_{1}\beta_{2}}\right]}^{2} \end{array}$$

Where, T is temperature in degrees C, β_1_ = 24.79°C d^−0.5^ and β_2_ = 0.525°C d^−0.5^.

The survivability coefficient, μ_*pi*_, of the pre-infectious particles (maturity < 1) started at one and decreased as a constant (−0.31 d^−1^) as long as the salinity was more than 30 ppt. When salinity dropped below 30 ppt, the survivability coefficient was calculated as:

(3) $$\begin{array}{r} \mu_{pi}\left(S\leq 30\right)=0.16\cdot S-5.11 \end{array}$$

Once particles molted (maturity ≥ 1), the survivability coefficient constant of −0.22 d^−1^. A constant survivability was chosen because there was not sufficient agreement among studies for the mortality rates of sea lice copepods at different temperature and salinity profiles (40).

The *μ*_*pi*_, was then used in the following exponential decay equation to calculate the survivability of each particle:

(4) $$\begin{array}{r} S_{\text{pi}}=\exp\left(\mu_{\text{pi}}t\right) \end{array}$$

### Sampling the particle tracking data

**Figure 2:**
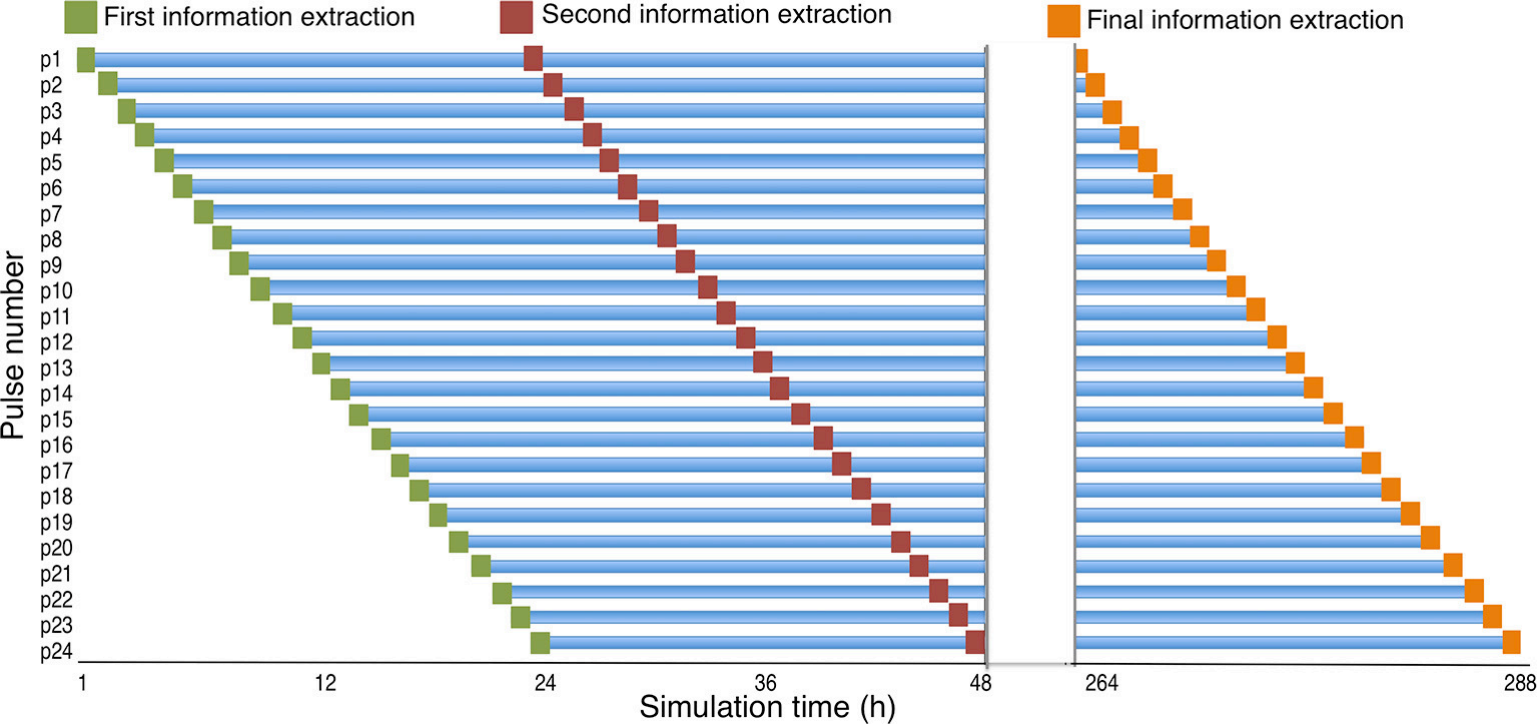
The cohort sampling scheme. The location of each “pulse” is extracted at their release, and every 24 hours after release until the particles are no longer tracked. Note that the x-axis (time) has been interrupted and omits hours between 49 and 264. This figure shows three of the 11 sampling points for a given cohort. These 11 sampling points, from each of 24 pulses on a given day, are combined to represent a cohort release day (CRD).

For this analysis, the focus was on the fate of sea lice larvae cohorts. We therefore defined one cohort of sea lice larvae as 24 h of pulses, or one day's worth of particle releases. Each cohort was followed over their lifetime by extracting the data and location for each particle every 24 h post release. Because pulses are released at intervals of 1 h, and each cohort consists of 24 h worth of pulses, there are 24 h of “real world” time between the initial and the final pulse for each cohort. Thus, by extracting information for each pulse at 24-h intervals following its release, we obtain a diagonal snapshot of data for each cohort that consists of 24 pulses of the same age spanning 24 h in simulation time (Figure 2). Because each pulse was followed for 11 days, there are 11 of these diagonal snapshots for each cohort. These 11 snapshots for each cohort were then combined into one dataset, and stored as a shape file for GIS manipulations. Because there were 141 days in the simulation, there were 130 such cohorts to follow over their lifetime, and each one is referred to hereafter as a “Cohort Release Day,” or CRD (Figure 2). The particles (*n* = 50) of each cohort were then separated by farm (*n* = 20) from which they were released, and their spatial position saved in 20 spatial point files of 13,200 points (50 particles/farm x 11 days x 24 h) for each CRD, resulting in 2,600 spatial point files (20 farms x 130 cohorts), to be used in the KDE process discussed below.

### Kernel density estimation

For each of the 130 cohorts, a KDE for the particles released from each farm (20) was constructed, and saved as a grid file (raster format) with a 100 × 100 m grid cell size. A bandwidth of 1/8 of the minimum extent of either the x or y coordinates (whichever was smaller) of the points was used. This was a compromise arrived at after trial and error to find an adaptive bandwidth that adequately smoothed the densities of the particles without being overly taxing in computational time. A polygon of the coastline was used as a bounding box for the KDEs. Diggle's edge correction was also used to control for bias introduced by the complex coastline. KDEs often spill over boundaries, and are thus susceptible to bias close to edges. If ignored, the bias can lower the value of the KDE near the origin (62).When the edge correction is used, the intensity value at point u is calculated as:

(5) $$\begin{array}{r} \lambda \left(u\right)=e\left(u\right)\sum_{i}k\left(x_{i}-u\right)w_{i} \end{array}$$

Where *k* is the Gaussian smoothing kernel, *e(u)* is an edge correction factor, and *w*_*i*_ are the weights.

The edge correction term *e(u)* is the reciprocal of the kernel mass (i.e., the absolute number of particles smoothed in the KDE) inside the window:

(6) $$\frac{1}{e(u)}=\;\int_{Wo}^{W}k\left(v-u\right)dv$$

Where, W is the observation window.

This KDE method took the biological model into account by weighting the KDEs on the survivability of the particles and filtering to include only particles with maturity ≥ 0.8. This value was chosen to ensure there were an adequate number of particles reaching maturity to be able to perform a KDE for each farm in each CRD. During times where the temperature was low in the simulation, no particles reached a maturity value of 1 (when they are deemed to become “infective”) for a number of farms. This may imply that our biological model for maturity was too sensitive to decreases in temperature. By setting the maturity threshold to 0.8, we mitigated this potential over-sensitivity to low temperature.

### Connectivity matrices

Every farm (*n* = 20) has its own KDE raster for each CRD (*n* = 130). The values in the KDE are expressed in particles per square kilometer, and represent the infestation pressure of that farm on the surrounding area. The value at the 100 × 100 m grid cell of the KDE raster where the farm of origin is located is defined as self-infestation. The values in the 100 × 100 m grid cells where each of the other 19 farms were located are assumed to represent the infestation pressure from the farm of origin. These values were extracted using the spatstat package in R. This information was stored in a 20 × 20 matrix, with emitting farms (“from”) on the x-axis, and receiving farms (“to”) on the y-axis, for every CRD. These matrices were then turned into a heatmap using ggplot in R, creating one heat-map per CRD in order to visualize clusters of farms and connectivity. The rows and columns in the heatmaps attempt to follow the approximate geographical arrangement of the farms from west to east. Note that the matrices are not symmetrical in that the infestation pressure from farm “a” to farm “b” is not necessarily equal in the opposite direction from farm “b” to farm “a.” The leading diagonal of the matrices shows self-infestation. The highest value of the heatmaps has been artificially set as 2 particles km^−2^, with any value above this being the same darkest purple as values of 2 particles km^−2^ in order to better illustrate detail at the lower connectivity levels.

### Network visualization

The infestation pressure from each site reaching all other sites was calculated for the entire 4.5 month simulation period by averaging the 130 [20 × 20] connectivity matrices, and was used to visualize the network of farms during this simulation period. To visualize consistent connections, as well as reduce small and potentially less biologically important connections (i.e., less likely for infectious larvae to attach to a host if the infestation pressure on a farm is very low), infestation pressures below 0.15 particles km^−2^ (two times the mean connectivity value) were not included in the visualization. Additionally, in order to capture a more typical network without the “peaks” in connectivity, CRDs 10–30 have been omitted from this analysis, as have CRDs 55–61, 90–95, and 120–125.

### Network dynamics

In order to visualize the network characteristics over the period under study, three analyses were conducted. First, the average connectivity across the entire network for each CRD was calculated by averaging all the values for each of the 130 matrices. The confidence interval for each average was calculated using boot-strapping methods. Second, the difference between the infestation pressure that each farm exerts and the pressure each farm receives, for a given CRD, was calculated using values from each connection matrix (values are in particles km^−2^). All the “from” values for each farm were summed into one “emitting” value (i.e., infestation pressure from a given farm on all the other farms). All the “to” values for a given farm, excluding self-infestation, were also summed into a separate “receiving” value (i.e. infestation pressure from all other farms on the given farm). A difference was then calculated based on these emitting and receiving metrics. If (received infestation pressure—emitted infestation pressure) > 3, the farm is considered a net receiver for that CRD. If the value was close to 0, the receiving/emitting infestation pressure was deemed to be balanced, while if (received infestation pressure—emitted infestation pressure) < −3, the farm was consider to be a net emitter for that CRD. Farms were considered “overall net emitters” for the simulation period if they had values < −3 for more than five CRDs during the simulation, or “overall net receivers,” if they had values > 3 for more than five CRDs. The +/– 3 threshold was chosen because it is twice the mean of the absolute value of received infestation pressure—emitted infestation pressure during the highest connectivity period (i.e., CRDs 11–30). This was to ensure it was capturing farms that truly where playing the role of net receiver or emitter.

Third, vertex betweenness scores for each farm were calculated for the network described in 2.9 using the “betweenness” command in the igraph package in r. Betweenness in this context refers to the number of shortest paths going through a vertex. The algorithm used to define betweenness of vertex v is defined in Brandes, 2001 (63) as:

(7) $$\Sigma (g_{i}v_{j}/g_{ij},i!=j,i!=v,j!=v)$$

### Software

All analysis was carried out in R (64). Net-CDF file manipulation was done using the “raster” package (65). Other data manipulation used the “tidyr” (66) and “dplyr” packages (67). GIS analysis used the “spatstat” package (68, 69). Graphics where created using the “ggplot2” package (70). Network analysis used the “igraph” package (71).

## Results

**Figure 3:**
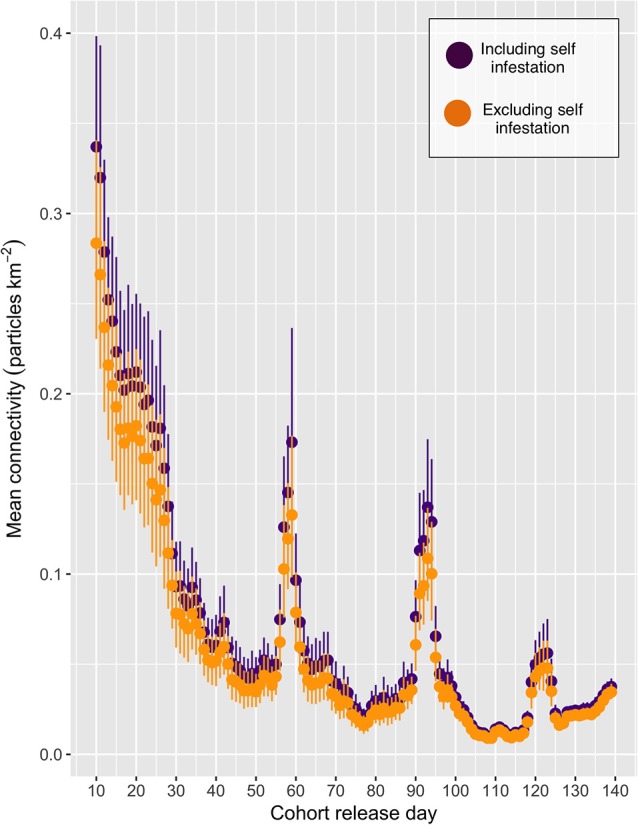
The mean connectivity for each cohort release day (CRD). The purple and orange points indicate the mean (+/- 95% confidence intervals) connectivity, including and excluding self-infestation, respectively.

The mean connectivity across the entire network over the period under study is shown in Figure 3, and indicates a decrease in the level of overall connection as the simulation progressed, with peaks at CRDs 59, 93, and 123. More than 99% of all particles remained within the model domain for all CRDs, with very few exceptions (particles where tracked regardless of mortality or maturity status).Results will focus on spatial patterns to determine “baseline” connectivity, in the absence of the stochastic events that may lead to spikes in connectivity, across the entire network for particular cohorts of particles.

**Figure 4:**
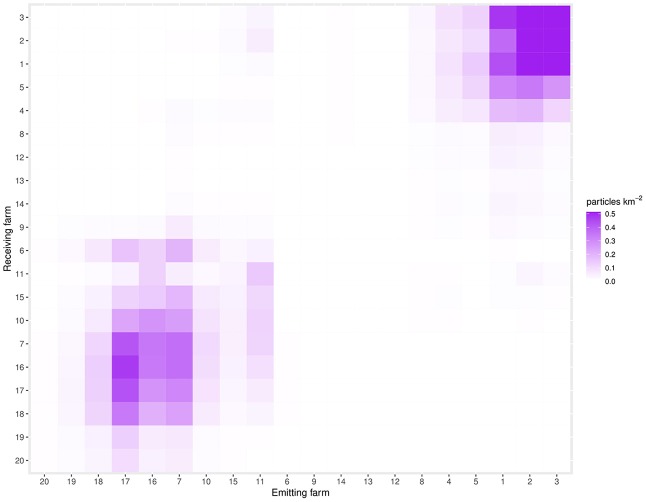
The connectivity matrix for CRD50. This CRD was chosen as being representative of an “average” day. The farms on the x-axis are emitting nodes (“from”), while the farms on the y-axis are the receiving nodes (“to”). Farms are ordered in approximate geographical order, from West to East. The diagonal from bottom left to top right indicates self-infestation.

A connectivity heatmap for CRD 50 is shown in Figure 4. This CRD was chosen as an example of a “typical” day (i.e., a CRD that is not a member of any connectivity peak). Additionally, a video made up of heatmaps for every CRD can be seen in the Supplementary Video. Values in the heatmaps represent the infestation pressure that a given farm (represented by each column) exerts on all other farms (where each is represented by a given row). Thus, the maps indicate a measure of connectivity between all possible pairs of farms (including between themselves; 20 farms × 20 farms = 400 possible connections). There appear to be two well defined sub-networks, consisting of farms 1–5 (sub-network A) and farms 7, 10, 15–18, which receive particles from farm 4 (sub-network B), both of which are often weakly connected to farm 11.

**Figure 5:**
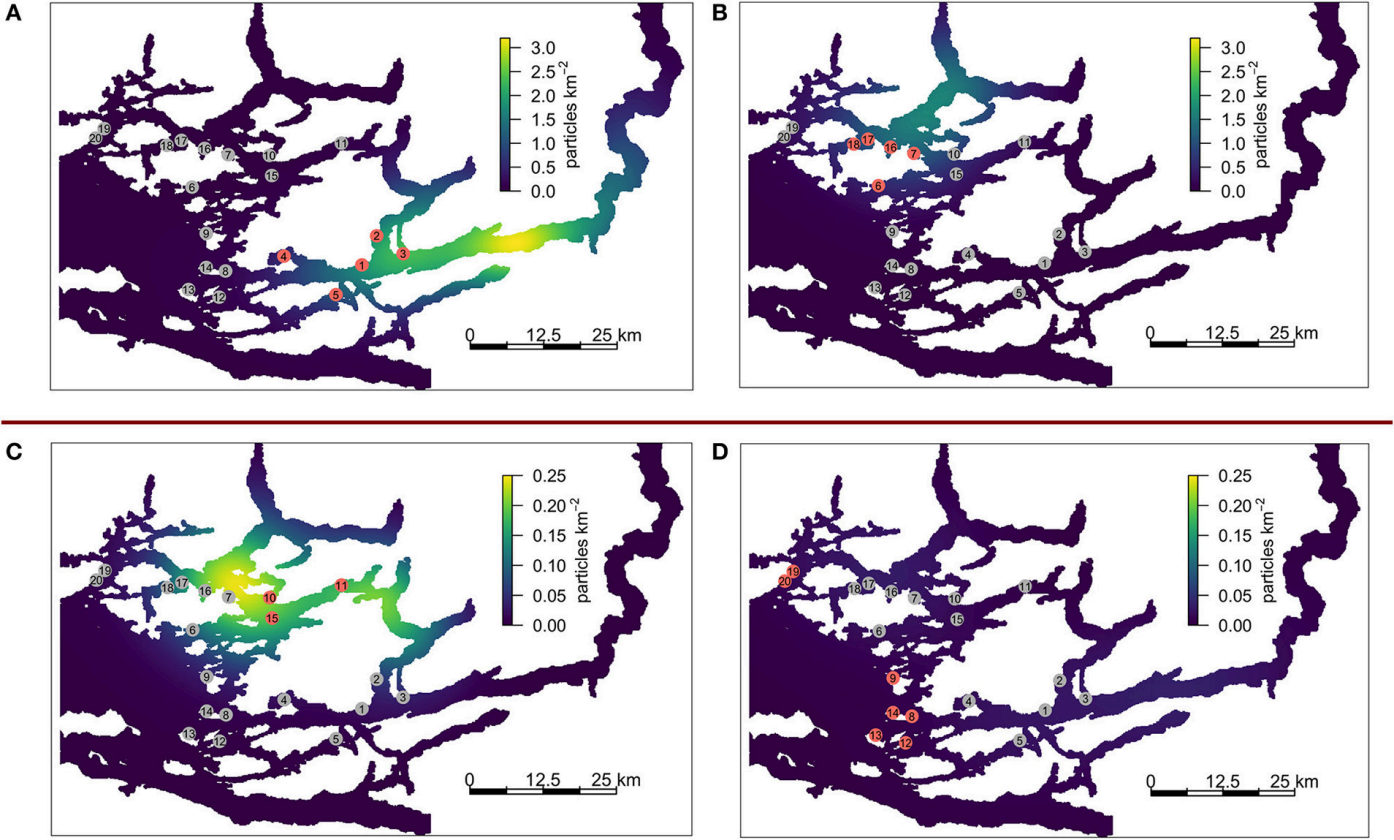
KDEs associated with various sub-networks for CRD50. **(A)** sub-network A, farms 1 – 5, **(B)** part of sub-network B, farms 6 – 7 and 16 – 18, **(C)** part of sub-network B, farms 10, 11, and 15 [which act as a bridge between sub-network **(A,B)**]. **(D)** Farms 8 – 9, 12 – 14, and 19 – 20 which are not strongly connected to either sub-network. Note that **(A,B)** and **(C,D)** are on different scales.

The KDEs for all farms on CRD 50 are shown in Figure 5, with each panel showing various groups of farms. All sub-networks exhibit the highest density of particles around 10 km or more away from the center of the contributing farms. The density centers for sub-networks A and B indicate that particles are frequently heading along the nearby channels in a north-easterly direction. In panel C (farms 10–11, and 15), the density center appear to be to the west of the contributing farms, with relatively few particles moving toward sub-network A. Panel D (farms 19–20, 8–9, and 12–14) indicates that these farms contribute very little to the overall network. Note that panels A and B are on a scale that is an order of magnitude higher than C and D.

The infestation pressure each farm experiences from all other farms (pressure received), minus the infestation pressure each farm exerts on all other farms (pressure emitted) over all CRDs is summarized in Table 1 (and shown in graphical format in Supplementary Material 1). These net infestation pressure metrics for each farm indicate the structural role each farm plays in the network of farms over time for this simulation period. No farms were net emitters or net receivers for the entire duration of the simulation, though farms 2, 11, 17, and 18 are receivers for much of the simulation. Only farm 4 crosses between being a “net emitter” and a “net receiver” over time.

**Table 1:** A summary of the role taken on by each farm in the network.

**Farm**	**# CRDs as an emitter > 3**	**# CRD's as a receiver < −3**	**Role in the network**
1	5	0	N
2	0	25	R
3	0	3	N
4	6	6	B
5	24	0	E
6	0	0	N
7	4	1	N
8	0	0	N
9	0	0	N
10	17	0	E
11	0	15	R
12	0	0	N
13	0	0	N
14	0	0	N
15	2	0	N
16	3	0	N
17	0	19	R
18	0	25	R
19	0	2	N
20	0	0	N

*Classification is based on the number of CRDs that a farm spends as an emitter, defined as (emitting pressure—receiving pressure) > 3, or as a receiver, defined as (emitting pressure—receiving pressure) < −3. Where either of these was the case for more than five CRDs over the course of the simulation a farm was classified as a “net emitter” (E), a “net receiver” (R), Farms where this was not the case for more than 5 days were classified as “neither” (N), while farm 4 was classified as “both” (B), as it functioned as a net emitter and receiver at different times during the simulation*.

**Figure 6:**
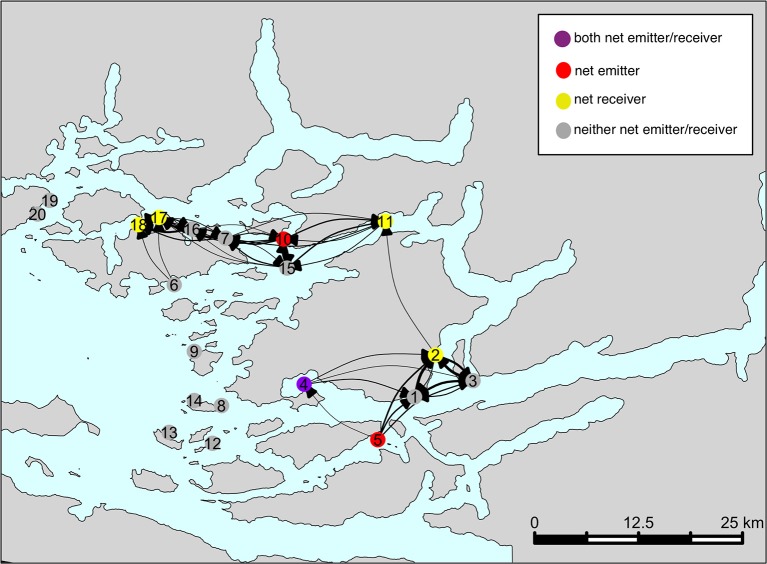
The typical network characterizing sea lice larvae exchange among farms in the BA. This network is based on connectivity estimates over the entire simulation period, excluding “peak” events.

A schematic representation of an “average” network is shown in Figure 6. The color of each farm indicates its structural role in the network (i.e. net emitter, net receiver, neither, or both) as determined in Table 1. This network does not include CRDs that were deemed to be “atypical” (i.e., CRDs 10–30, and the three peaks around CRD 59, 93, and 123), in order to capture the baseline connectivity network. The sub-networks identified in the Figure 4 are also seen in the network shown here. Farms 1–5 are connected (“sub-network A”); as are farms 6–7, 10, and 15–18 (“sub-network B”). Sub-networks A and B are weakly connected through farm 11. Farms 8, 12, 13, 14, 9, 19, and 20 appear to have low connectivity to other sites in terms of sharing infective sea lice larvae.

The betweenness scores are summarized in Table 2. These show farm 11 with the highest betweenness score, and farms 2, 7, and 15 also having high scores (between 15 and 30). Farms 16, 17, and 18 also show non-0 betweenness scores, though much lower (< / = 5).

**Table 2:** Farm vertex betweenness scores.

**Farm**	**Betweenness**
1	0
2	29
3	0
4	0
5	0
6	0
7	20
8	0
9	0
10	0
11	30
12	0
13	0
14	0
15	14
16	5
17	4
18	1
19	0
20	0

## Discussion

One of the biggest challenges in quantifying connectivity based on bio-physical modeling of marine systems is the question of how best to capture the different amounts of time particles spend in each receiving location. The most commonly used approach in the sea lice literature draws a polygon around a receiving area, and counts each time a particle enters the polygon. The particle can then be removed from the simulation (9), or allowed to continue (7, 15, 20). Continuing particles can either be counted once per time it enters the receiving area (20) or multiple times if it remains in the receiving area (35). These approaches have been used in connectivity modeling of sea lice in salmon farming regions such as Scotland, Norway, and the Faroe Islands (7, 9, 11, 20, 72). However, in the case of infectious agent networks, infection or infestation is a result of IP, that is, exposure time and infectious dose. Therefore, a particle that passes through a polygon in less than an hour does not exert the same IP as a particle that spends 3 days within the polygon. This is partially addressed by the method used by Samsing et al. (7), which allows multiple connections to be counted when a particle spends an extended amount of time within a receiving polygon. The use of KDE is a more holistic approach because it incorporates both time and space into the connectivity estimates of infectious agent networks.

We apply this method to provide insights toward infectious agent connectivity in aquatic environments in the context of farm-to-farm sea lice infestation. Controlling sea lice is one of the most important health management considerations on Atlantic salmon farms and for minimizing farm impacts on native salmonid populations. Because sea lice can be transported in water currents upwards of 30 km (7, 20), and can be transported for several days before molting into an infectious stage, IP will not necessarily decrease with increasing distance from release sites, as could be expected for other marine pathogens such as viruses that spread directly through water (73). Therefore, bio-physical models can potentially produce far better estimates for sea lice larval modeling (assuming model assumptions are realistic), in order to adequately characterize sea lice networks. Given the complexity of problems surrounding sea lice control globally, such as the development of populations resistant to treatments and the high cost of administering treatments (26, 52, 74–76), spatial planning considering sea lice epidemiology and larval dispersal capabilities is crucial for the sustainability of the industry.

However, it is worth noting that it is not yet clear exactly how the theoretical IP that farms experience from neighboring farms translates into sea lice loads, on either the salmon in the farms or the wild salmon migrating through the KDE “clouds.” There have been a few attempts at quantifying this using lab experiments, mathematical models, and field studies (48, 77, 78), but wide disagreement across the field still exists. These need to be resolved in order to assess in absolute terms how large a threat to wild salmon or neighboring salmon farms the IP measures from our studies might be. However, even without this resolution, we can assess relative risk to each other (e.g., farm 4 has a larger IP impact on its neighbors than the rest of the farms in sub-network A), and describe the network based on IP estimates from our models to support hypothesis generation around management recommendations. Once the model is validated, these recommendations could potentially be implemented. While not a formal validation, it is worth noting that other papers in the area have found similar large-scale spatial relationships. Rees et al. (29) found general patterns similar to those in this study, using a GLM approach to link farm and wild salmon infestation levels. The highest probabilities (and intensities) of infestation occurred near the farms in the inlets on the BA in areas we found farms to be highly connected, with lower probabilities and intensities found near the farms closer to the open ocean. These farms we found to be not highly connected to other farms. Farm 6 (in our study) is particularly noteworthy, as it is near the open ocean but the area around it still had a high probability of infecting wild salmon in Rees et al. and we found it to be highly connected to sub-network 2 (29). Patanasatienkul et al. (25), found evidence of clusters of elevated sea lice infestation on wild pink and chum salmon in the BA, with three distinct clusters of non-motiles (i.e., more recent infestations) which locations are similar to the locations of the sub-networks of farms we found (i.e., cluster 1 corresponds spatially to sub-network B, cluster 2 to sub-network C, and cluster 1 to sub-network A) (25). Additionally, for diseases where this is better understood, these absolute values could potentially be translated into predictions around the likelihood of disease (79).

### Drivers of connectivity variability

Connectivity via living organisms (i.e., larvae, gametes, etc.) is ultimately the result of a complex combination of physical and biological drivers. Temporal connectivity variability, such as seen in Figure 3, could be caused by changes in physical and biological drivers, or more likely, the combination of both. Changes in winds from storms, temperature drops in fjordic areas from increases in river discharge, or in more open-ocean areas from upwelling events are just a few examples of physical changes that can drastically alter circulation patterns in the short term (10). Although identifying the drivers of the peaks in connectivity seen in Figure 3 is beyond the scope of the present study, the strong relationship between water temperature and particle maturity (40) as well as water circulation (11, 13) emphasizes the potential bio-physical models have to inform connectivity models. A further study will explore in depth the relative contribution of physical and biological drivers on sea lice connectivity for this simulation in order to explain the exponential decrease seen in the first 30 CRDs, as well as the spikes around CRDs 59, 93, and 123 (Burnett et al., in preparation). It is, however, likely tied to salinity and temperature fluctuations related to the spring freshet, when large amounts of fresh and cold water enter the BA from the surrounding rivers.

### Sea lice connectivity among salmon farms of the BA and management implications

Over our simulation period, we found evidence that the farming region had two main areas of connectivity, and that these were linked by farm 11 (Figure 6). This was also confirmed by the high betweenness score of farm 11 (the highest of all farms, with a score of 30). Consequently, investigation efforts should be made to see whether additional sea lice treatment and surveillance effort should be focused on this site to break parasite transmission between the two sub-networks of farms. Caution may be warranted in the case of any future applications for new farm sites along the Tribune Channel, where the farms already located near here (i.e., 11, 2, 7, and 15) had the highest betweenness scores of the network as it may strengthen the connection of the two sub-networks and could potentially lead to more difficult sea lice control. Farms located further from channels and more toward the open ocean would theoretically be a better location from a sea lice management perspective, given the lack of connectivity detected among these farms, and betweenness scores of 0 (i.e., 8, 9, 12, 13, and 14). It is likely that these farms had a low connectivity on their neighbors because their simulated sea lice larvae are being simply flushed out of the channel by the time they reach maturity and become infectious. Counter-intuitively, while the rest of the tribune channel farms (i.e., 2, 11, 15, and 7) had high betweenness scores, farm 10 had a score of 0, despite being classified as a net emitter. This is likely due to some sheltering affect of farm 10 being placed far enough into the bay that sea lice moving through this channel do not infect farm 10. Thus, it is not receiving particles from many neighbors (thus lowering its betweenness score), but is emitting its particles to the rest of sub-network B.

In Figure 3 the confidence intervals of the points with self-infestation overlap with those that include no self-infestation, indicating that self-infestation does not play a large role in this particular model system. This coupled with Figure 5 demonstrating that the location with highest density of particles can be tens of km away from the emitting farms indicates that the particles are being carried away before they mature and reach infectiousness. Thus, farms in the BA would likely benefit from coordinated treatment efforts, particularly within sub-network A and B. Without regional coordination with treatments, re-infection after treatment is likely from farms in the sub-network that have not also been treated. In addition, connectivity measurements based only on seaway distances, as can be found in a number of studies in the literature, may inadequately describe the transmission patterns between farms. However, until the bio-physical model is validated, these sub-networks of farms remain only a tool for hypothesis generation for field tests. Additionally, sensitivity analysis of simulation parameters, particularly around the biological model (i.e., maturity and mortality) should be conducted, as small changes in these parameters have the potential to have a large impact on connectivity scores. These studies are planned in forthcoming papers.

It is worth noting that simulation parameters were based on studies of the Atlantic *L. salmonis salmonis* subspecies, and the BA has *L. salmonis oncorhynchi* subspecies. Morphological and biological differences between the subspecies have been documented (80). However, these parameters were the most well studied available at the time of running the simulation, so were used despite the possibility of subtle differences between subspecies.

Additionally, though there is evidence of burst swimming behaviors in sea lice, such as salinity avoidance, temperature seeking, host finding, and diel vertical migrations (81), these were not included in the model. This is because while the burst swimming abilities of sea lice appears to be important for host finding in short distances, there is conflicting evidence on if burst swimming plays an important role in large-scale dispersal (55, 82–84). Johnsen et al. (85) found that the light sensitivity behavior in sea lice did not have a large effect in model outcomes. They did, however, find that vertical movements could impact horizontal dispersion, which in our case could impact connectivity. However, they also found that even with different swimming speeds and turbulence response behaviors, the greatest densities of lice were always found at the surface. Additionally, laboratory experiments have shown different swimming behaviors between copepods and nauplii (86). Given this uncertainty around biologically appropriate behaviors, and the fact that even with different behaviors/ parameters (i.e., swim speeds and turbulence avoidance) the greatest densities of sea lice were found at the surface in the Johnson simulation, we chose not to include this in our model. However, this potential model limitation should be kept in mind.

### Strengths and weaknesses of the KDE approach

The approach described in this paper of using KDEs to estimate the IP each farm experiences from the other farms, has several strengths, particularly in regards to quantifying infectious agent networks. Firstly, estimating the IP the network nodes (in this example, farms) experience from their neighboring nodes incorporates both exposure time and infectious agent (i.e., particles) density, and therefore is more relevant for infectious agent networks than other measures of connectivity, such as probability of connections. The method can be applied to any aquatic system with transmission through water, in which networks connected by infectious agents emitted from point sources are of interest. Some marine disease outbreaks that have had large economic and ecological consequences and are good candidates for having this more holistic method of quantifying infectious agent networks applied include: sea star wasting disease in the Pacific Northwest, infectious withering system in California Black Abalone, and sea grass wasting disease in the Atlantic, etc. (87). Additionally, because the locations of the particles across their lifetime are smoothed in KDEs, this method can be applied to fairly temporally dispersed particle tracking data, so it is not necessary to record the location of every particle every few minutes. This lower temporal resolution requirement can significantly reduce computational demands and processing time. Additionally, since the output is a smoothed surface estimating particle densities for the entire region, it could be used to simulate specific outbreak scenarios across a region by scaling the number of particles released from each farm/node based on the biomass of the fish stock at each farm.

Weaknesses around the use of KDEs to estimate IP are, firstly, knowing what impact varying levels of IP has on organisms in receiving nodes/farms. Translating IP experienced by a farm into sea lice counts on farmed salmon or wild salmon passing near a farm (or onset of disease, in the case of other infectious agents) is the domain of experimental lab and field studies (54). Secondly, the simulation in this paper does not model settlement or recruitment processes, which are critical aspects of larval connectivity and can be influenced by temperature and salinity (88). Including these model extensions into the bio-physical model should yield more accurate estimates of IP on the farms, as the IPs generated by the model would reflect the likelihood of infectious sea lice larvae settling onto a host salmon.

## Conclusions

In summary, we utilized output from a bio-physical model for a kernel density estimation (KDE) approach in order to quantify connectivity of 20 salmon farms in the Broughton Archipelago during a critical 4.5 month period (March 2nd–July 30th) when juvenile wild salmon are out-migrating to the ocean. The KDE approach allowed us to take both time and space into account when making the infestation pressure estimates each farm exerts on the others. These methods incorporate climatology, space, time and the biology of the infectious agent (in this case, the sea louse) into connectivity estimates. In areas where accurate connectivity information is important for management decisions and conservation efforts, incorporating coupled bio-physical models with KDEs could be a useful tool. This is particularly true for infectious agent networks, where understanding infection or infestation pressure each source of particles exerts on the region is crucial.

Understanding these sea lice larvae networks may support future advice for best treatment practices, generate hypotheses for future empirical studies in the BA, and provide a better understanding of sea lice transmission patterns in the Broughton Archipelago. This can lead to improved marine spatial planning to mitigate sea lice transmission, and could ultimately contribute to an increasingly sustainable salmon farming industry in British Columbia, Canada. Furthermore, the approach demonstrated in this paper can be applied to other aquatic scenarios where meta-populations are connected via infectious agents for improved spatial planning, treatment strategies, and hypotheses generation around preventing or mitigating outbreaks.

## Data availability statement

The raw data supporting the conclusions of this manuscript will be made available by the authors, without undue reservation, to any qualified researcher.

## Author contributions

All authors contributed to study design, ideas for analysis, discussion of results and editing of the manuscript. DC carried out all analysis and figure generation and wrote the manuscript with input from all authors. ER ran the initial particle tracking simulation. CR supervised the project.

### Conflict of interest statement

The authors declare that the research was conducted in the absence of any commercial or financial relationships that could be construed as a potential conflict of interest.
